# Usability Assessment and Task Performance of Health Application for Older Adults:

**DOI:** 10.1093/geroni/igaf122.2814

**Published:** 2025-12-31

**Authors:** Suhyun Kim, Jungyeon Bae, Dahyeon Lee, Eunseo Lee, Yunseo Jang, Daeun Jeong

**Affiliations:** Kyungpook National University, Daegu, Republic of Korea; Kyungpook National University, Daegu, Republic of Korea; Kyungpook National University, Daegu, Republic of Korea; Kyungpook National University, Daegu, Republic of Korea; Kyungpook National University, Daegu, Republic of Korea; Kyungpook National University, Daegu, Republic of Korea

## Abstract

**Purpose:**

This study aimed to evaluate the usability and task performance of health applications for older adults to identify ways to enhance user experience.

**Methods:**

This study utilized an explanatory sequential mixed methods approach. The quantitative phase involved a survey of 100 older adults from senior welfare centers in D City utilizing the Mobile Application Rating Scale (MARS) to assess app usability. The qualitative phase involved in-depth individual interviews and task performance evaluations to explore functional challenges and usability barriers.

**Results:**

The overall MARS score was 3.56 out of 5, with functionality receiving the highest rating (3.60), followed by aesthetics (3.57), information (3.78), engagement (3.71), and subjective quality (3.16). The mean task performance score was 2.09 out of 3, with significant obstacles identified in app installation, information retrieval, navigation complexity, and input processes. The interviews revealed usability challenges, such as insufficient font size, lack of screen magnification, and unintuitive interface designs. Participants emphasized incorporating practical functions and in-app user guidance to enhance usability.

**Conclusion:**

Nurses can help ensure that health applications are tailored to older users’ needs. Improved app usability for this population requires design modifications, including adjustable font sizes, screen magnification options, and intuitive interfaces. Additionally, user education and in-app user guides can support effective app usage and task performance.

